# Atmospheric Pressure Plasma Jet Exposure of Polylactic Acid Surfaces for Better Adhesion: Plasma Parameters towards Polymer Properties

**DOI:** 10.3390/polym16020240

**Published:** 2024-01-15

**Authors:** Andrei Vasile Nastuta, Mihai Asandulesa, Florica Doroftei, Ioan-Andrei Dascalu, Cristian-Dragos Varganici, Vasile Tiron, Ionut Topala

**Affiliations:** 1 Physics and Biophysics Education Research Laboratory (P&B-EduResLab), Biomedical Science Department, Faculty of Medical Bioengineering, “Grigore T. Popa” University of Medicine and Pharmacy Iasi, M. Kogalniceanu Str., No. 9–13, 700454 Iasi, Romania; 2 “Petru Poni” Institute of Macromolecular Chemistry, 41A Gr. Ghica Voda Alley, 700487 Iasi, Romania; asandulesa.mihai@icmpp.ro (M.A.); florica.doroftei@icmpp.ro (F.D.); idascalu@icmpp.ro (I.-A.D.); varganici.cristian@icmpp.ro (C.-D.V.); 3 Research Center on Advanced Materials and Technologies (RAMTECH), Department of Exact and Natural Sciences, Institute of Interdisciplinary Research, “Alexandru Ioan Cuza” University of Iasi, Blvd. Carol I No. 11, 700506 Iasi, Romania; vasile.tiron@uaic.ro; 4 Iasi Plasma Advanced Research Center (IPARC), Faculty of Physics, “Alexandru Ioan Cuza” University of Iasi, Blvd. Carol I No. 11, 700506 Iasi, Romania; ionut.topala@uaic.ro

**Keywords:** atmospheric pressure plasma jet, adhesion, plasma treated polymer surface, surface characterization, atomic force microscopy

## Abstract

Polymers play a crucial role in multiple industries; however, surface modification is necessary for certain applications. Exposure to non-thermal plasma provides a viable and environmentally beneficial option. Fused deposition molding utilizes biodegradable polylactic acid, although it encounters constraints in biomedical applications as a result of inadequate mechanical characteristics. This study investigates the effects of atmospheric pressure plasma generated by a dielectric barrier discharge system using helium and/or argon on the modification of polylactic acid surfaces, changes in their wettability properties, and alterations in their chemical composition. The plasma source was ignited in either He or Ar and was tailored to fit the best operational conditions for polymer exposure. The results demonstrated the enhanced wettability of the polymer surface following plasma treatment (up to 40% in He and 20% in Ar), with a marginal variation observed among treatments utilizing different gases. The plasma treatments also caused changes in the surface topography, morphology, roughness, and hydrophilicity. Plasma exposure also resulted in observable modifications in the dielectric characteristics, phase transition, and structure. The experimental findings endorse the utilization of plasma technologies at normal air pressure for environmentally friendly processing of polymer materials, specifically for applications that necessitate enhanced adhesion and have carefully selected prerequisites.

## 1. Introduction

Polymers are crucial in numerous sectors, such as biology, medicine, various other industries (including construction, textiles, and machinery), and more recently, the food industry. However, there are some situations where it is necessary to modify the surface properties of polymers before proceeding with additional processing. This is due to the often inadequate surface characteristics of polymers for certain applications. Due to their predominant bulk physicochemical properties, these materials are mostly used as essential components in a diverse range of mechanical, electrical, or biocomponents. Nevertheless, in numerous applications, the surface characteristics of the polymers take precedence, necessitating meticulous focus on both finding and achieving these qualities. The usage of non-thermal plasma exposure is a procedure that is widely utilized for the purpose of modifying the surface properties of polymers [1,2,3,4].

One of the most commonly studied properties of plastic materials is the surface adhesion, which is studied by means of wetting the surface and determining the work of adhesion. The presence of good wetting does not, on its own, result in good adhesion. In order to do this, the surface free energy of the plastic is frequently enhanced by the process of pre-treatment. Plasma discharges, corona or flame treatment, and the action of oxidizing gases like ozone or fluorine are some of the common procedures that are utilized in this context [5,6,7,8,9,10,11].

The use of atmospheric pressure plasmas is a practical and ecologically friendly alternative to the conventional physicochemical methods that are currently being utilized in the field of technology for the purpose of material interaction. The plasma–matter interaction is characterized by a high concentration of reactive nitrogen and oxygen species (RNS/ROS) due to the numerous chemical and physical processes that take place during this interaction. In addition to this, they are an indispensable component in a wide range of sectors and professions, including those that deal with food, environment, agriculture, healthcare, materials science, and even the automotive industry. Numerous challenging research projects that involve multiple disciplines are concentrating their efforts on the intriguing new applications of plasma–surface, plasma–liquid, and plasma–gas interactions. Biomedicine (as an antibacterial, disinfectant, antiseptic, wound healing promoter, and selective cancer cell and tumor treatment agent), pharmacology, food science, bioengineering, agriculture (as an additive to green fertilizer or to help seeds germinate), and transportation are just some of the many fields that could potentially benefit from plasma’s wide range of applications. Having stated that, the technologies that are based on plasma are the plasma directions that are being discussed above [12,13,14].

Therefore, it is crucial to acquire a comprehensive understanding of polymers by gathering a substantial amount of knowledge. Despite the multitude of published experimental results, the exact processes of plasma–polymer surface interactions have not been fully understood, making it a current area of interest. Considering this, it is crucial to acquire a comprehensive understanding of polymers by gathering as much information as possible. Many other features of polymers can be adjusted, including surface morphology, surface energy, surface flexibility, coefficient of friction, and interfacial tension coefficient. Hence, the need to have knowledge of all these parameters prompts the invention and optimization of the novel physico-chemical techniques capable of altering them. As previously stated, subjecting polymers to atmospheric pressure non-thermal plasma can be a viable method for altering the surface and manipulating the structure through physical or chemical processes such as cleaning, etching, crosslinking, functionalization, and even polymerization [4,15,16,17]. The operating characteristics of plasma, such as applied voltage, discharge current density, electrical mean power, total power of emitted radiation, and excited and reactive species, have an impact on the primary processes occurring at the interface between plasma and polymer. Hence, by manipulating these parameters, various plasma-induced changes can be observed on the polymer’s surface. Typically, as a result of atmospheric pressure conditions, the surface has a large amount of chemical groups that contain oxygen, such as hydroxyl, carbonyl, or carboxyl/ester, even if the plasma working gas used was not oxygen, but rather air, carbon dioxide, helium, or even argon. The abundance of oxygen-containing functional groups is typically attributed to the strong attraction of oxygen to the polymer surface exposed to plasma.

Understanding the plasma–polymer surface interactions is crucial for a comprehensive understanding of polymers’ behavior. Subjecting polymers to atmospheric pressure non-thermal plasma can alter the surface and manipulate the structure through physical or chemical processes. Operating characteristics of plasma, such as applied voltage, discharge current density, electrical mean power, total emitted light, and excited and reactive species, impact the primary processes occurring at the interface between plasma and polymer.

In the field of material engineering, the fabrication of three-dimensional scaffolds is a common use of additive manufacturing (AM) techniques. Some examples of these processes are stereolithography, fused deposition molding (FDM), and selective laser sintering (SLS). Extrusion-based printing, often known as FDM, is the additive manufacturing technology that is utilized most frequently in sectors such as electronics, architecture, and tissue engineering. Polylactic acid (PLA), a polymer that is both biodegradable and renewable, has garnered attention due to the fact that it is biocompatible, can be easily processed, and has a low level of toxicity. On the other hand, its limited use in biomedical applications is hindered by its poor mechanical qualities, insufficient surface wettability, and slow degradation rate. Improvements in implant mechanical qualities and biocompatibility can be achieved using a variety of methods, including the use of fillers made of natural polymer, ceramic, or metallic materials, surface modification techniques, and coating.

Because 3D printing allows for the construction of complicated structures with unmatched accuracy, it has revolutionized the manufacturing business. While designing materials for use in 3D printing processes, the consideration of the contact angle is essential. The surface tension and wetting properties of printing materials impact their adhesion and stacking, which in turn affect the quality and integrity of the final product. Thanks to developments in 3D printing spurred by research into contact angles, a wide range of businesses may now more easily produce unique and intricate designs.

In this investigation, the effects of atmospheric pressure plasma generated by a dielectric barrier discharge (DBD) system operating in He and/or Ar on the surface modification, wettability characteristics, and the chemical composition of polylactic acid are examined.

## 2. Materials and Methods

This section consists of information about the materials and methods that were used in this study. The section is divided into two parts: one related to the experimental arrangement and methods used for plasma source ignition, characterization, and treatment—Section 2.1; and the second includes the pristine polymers (polylactic acid, PLA, filaments, and the 3D-printed PLA samples) and those exposed to plasma treatment as well as the physico-chemical methods used for samples characterization—Section 2.2.

### 2.1. Plasma Source and Electro-Optical Diagnosis

**Figure 1:**
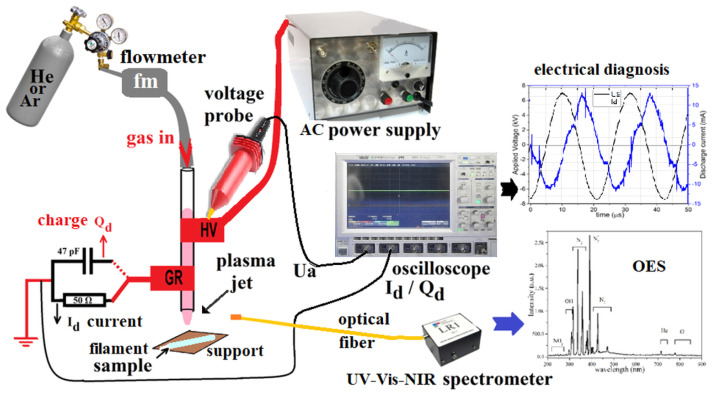
The experimental arrangement employed in this work consists of the plasma source, as well as the electrical and optical diagnostic devices.

An atmospheric pressure plasma jet device is used to treat commercial polymer fiber for 3D printing, as shown in Figure 1. In these studies, He and Ar were used as feed gases that were flowing through the discharge tube (made of quartz, 100 mm in length, with an inner diameter of 4 mm and an outer diameter of 6.1 mm) at a flow rate of 2.0 standard liters per minute. The high voltage and ground electrodes, made out of 10 mm wide and 0.5 mm thick commercial adhesive copper tape, were wrapped on the exterior of the quartz tube at a gap of 10 mm, the ground one being 5 mm away from the tube edge. A 5 mm gap between the discharge tube and the polymer sample surface was maintained during the experiments. The power supply generates bipolar AC output with the peak voltage of 0–20 kV at a frequency of 48 kHz. The discharge power was calculated via Lissajous figures formed with the charges across the capacitor and the applied voltage across the discharge. A similar arrangement was previously used by Huzum, Nastuta, and Burducea for the treatment of grape must, for plasma parameter optimization, and for wheat seed treatment [18,19,20], attaining an appropriate set of plasma settings for sample exposure under investigation.

Throughout the course of these investigations, all plasma treatments of polymeric materials have been carried out by employing the atmospheric pressure plasma jet at an applied voltage of maximum 18 kV. This has resulted in a discharge mean power of up to 10 W.

In this experiment, one electrode was linked to the power cord of the power supply, namely the high voltage electrode (HV). The second electrode was connected to the ground, serving as the grounded electrode (Gr). Both electrodes were wrapped around the outer surface of the quartz tube, following a similar approach as that previously described in references [18,21,22,23]. The discharge was initiated using a power supply (PVM500, Information Unlimited, Amherst, MH, USA) capable of generating a voltage range of 1 to 40 kV, a frequency range of 20 to 70 kHz, and an adjustable power output of 10 to 300 W. This power supply allowed for the independent control of voltage, current, and frequency. The applied sinusoidal voltage Ua (up to 18 ${\mathrm{kV}}_{peak-to-peak}$ , at a repetition rate of 48 kHz) and the total current of the discharge Id, were monitored using voltage and current probes (high-voltage probe Caltest CT4028, Cal Test Electronics Inc. (Yorba Linda, CA, USA), voltage probe Lecroy PP006A (Chestnut Ridge, NY, USA), wideband current monitor Pearson 150 (Palo Alto, CA, USA), 50 Ω charge resistor, a 47 pF charge capacitor), and a 1 GHz digital oscilloscope (Lecroy WaveSurfer 104Xs, four channels, 2.5 GS/s, Chestnut Ridge, NY, USA). The experimental setup involved the utilization of pure helium and pure argon (He 5.0 and Ar 5.0, Siad Romania, Bucharest, Romania) as the working gases. These gases were introduced into the discharge quartz tube at a constant flow rate of 2.0 standard liters per minute (slm) using a needle valve rotameter (0–5 slm Platon NGVS312 series, CT Platon, Saint Etienne, France). The spectral emission of the discharge in the ultraviolet-visible-near-infrared (UV-Vis-NIR) range of wavelengths from 200 to 1100 nm was analyzed using an LR1 broad range spectrometer (ASEQ Instruments, Vancouver, BC, Canada). The spectrometer consisted of a monochromator with a 50 μm entrance slit, a 600 gr/mm diffraction grating blazed at 300 nm, and a CCD (Toshiba TCD1304DG linear array) detector. The spectrometer was connected to a 0.4 mm diameter and 1 m long kevlar-reinforced and cosine-corrected optical fiber (Thorlabs, Newton, NJ, USA), which was designed for wavelengths ranging from 200 to 1200 nm. It was positioned at a distance of 5 mm from the plasma.

Both untreated and plasma-treated versions of the polymer were employed in the tests. One set of samples was comprised of the polymer filament before and after being subjected to the plasma treatment, while the other set consisted of polymer that had been printed using 3D technology. For this particular study, commercially available white polylactic acid filament with a diameter of 1.75 mm ( ±0.05 mm, from Devil Design Sp. J., Milkolov, Poland) was utilized. Several types of shapes were used for the analyses of 3D-printed PLA samples. More precisely, for surface analysis, FTIR, and XRD analysis, besides PLA filaments, cuboid objects with a base of 10 × 10 mm and a height of 0.5 mm were printed. For the dielectric analyses, cylinders with a radius of 10 mm and a height of 0.5 mm were prepared. A Prusa i3 MK3S+ 3D printer (Prusa Research a.s., Prague, Czech Republic) based on fused deposition modeling (FDM) technology was used for these experiments. The printing parameters, based on the printer’s manufacturer and PLA filament producer, were the nozzle temperature at 210 °C, with a heated bed at 60 °C, using a 0.15 mm layer height (quality setting from the PrusaSlicer software, v2.6.0), and a 100% rectilinear infill.

### 2.2. Surface and Volume Polymeric Sample Characterization Methods

Despite the fact that a significant number of PLA’s mechanical, electrical, and thermal properties have been thoroughly investigated, there is a paucity of information available in the literature regarding the surface properties of this material. Nevertheless, new applications, such as in the processes of 3D printing for a variety of applications, necessitate the development of novel surface qualities, which involve the biocompatibility of the material. Considering that the protection of the environment has emerged as a leading concern for our contemporary civilization, altering the surfaces of these materials in plasma appears to be a potentially useful approach.

In order to further investigate the modifications induced by exposing the polymeric samples to the discharge, several surface and volume characterization methods were used.

#### 2.2.1. Surface Morphology: Atomic Force Microscopy and Scanning Electron Microscopy

Atomic force microscopy (AFM) is a highly effective technique utilized for the analysis and evaluation of polymers at the nanoscale. This technique has the capability to offer valuable insights into the surface morphology, topography, elasticity, and adhesion properties of polymers. Atomic force microscopy (AFM) is employed for investigating several phenomena in polymer science, such as the phase separation characteristics of polymer blends, the kinetics of crystallization in semicrystalline polymers, and the interactions between polymers and other materials. Atomic force microscopy (AFM) is a powerful technique that enables the visualization and analysis of the surface morphology and topography of polymers with exceptional precision and detail. The provided data can be utilized for investigating the impact of processing parameters, such as plasma treatment, on the surface morphology of polymers [24,25,26]. Additionally, it can aid in the identification of flaws and impurities.

Atomic force microscopy was involved in order to investigate, at the nanometric scale, the particularities of both pristine and plasma treated samples: the topography images and roughness parameters of the surface. Therefore, a Solver Pro-M atomic force microscope (AFM, NT-MDT, RU) in tapping mode, in air, equipped with an NSC21 cantilever (standard n-type silicon phosphorus doped, triangular tips, tip curvature radius <10 nm, force constant = 17.5 N/m, resonance frequency = 201 kHz, Mikromasch, Sofia, Bulgaria) was used.

Scanning electron microscopy (SEM) is an effective polymer characterization tool. This method can magnify polymer surface morphology, microstructure, and content up to one million times. SEM is useful for studying polymers’ nanoscale qualities, which often determine their attributes and performance. In polymer characterization, scanning electron microscopy is often used to study surface/cross-section morphology. This method can identify and characterize cracks, voids, inclusions, and polymer mix and composite morphology. SEMs are useful for studying polymer surface topography. SEM is used in polymer characterization, especially microstructure analysis. This method can be used to identify phases in polymer blends and composites, and to study crystalline polymer morphology. SEM may also investigate polymer chain orientation, which is important in fiber spinning and film processing.

For the present study, the SEM investigations were realized on the cross-section of PLA 3D-printed objects, before and after mechanical stretching. The investigated samples were coated with a thin layer of platinum using a Leica EM ACE200 Sputter coater to render electrical conductivity and to obstruct charge build-up during exposure to the electron beam. The samples were fractured and the polymer cross-sections were examined on a scanning electron microscope Verios G4 UC (Thermo Fisher Scientific, Waltham, MA, USA), at an accelerating voltage of 5 kV using an Everhart–Thornley SE detector.

#### 2.2.2. Static Contact Angle and Surface Energy

The characterization of wettability in polymers involves evaluating two parameters: static contact angle and surface energy. These measurements are crucial in investigating the impact of surface alterations on the wettability of polymers, forecasting adhesion behavior between polymers and other surfaces, and examining the adsorption phenomena of liquids and proteins on polymer surfaces [8,27,28,29]. The static contact angle, which is the angle between the tangent of a liquid droplet and the surface of a solid material at the specific place where they come into contact, is a valuable technique for assessing the wettability characteristics of a polymer surface. A high contact angle indicates hydrophobicity, while a low contact angle indicates hydrophilicity. Surface energy—the amount of energy required to generate a fresh unit area of surface—is determined through the measurement of static contact angles shown by two liquids possessing distinct surface tensions. These measurements can serve as predictive tools for various characteristics, including wettability, adhesion, and adsorption. The objective of this study is to create novel polymer materials with specific wettability and adhesion characteristics.

The surface energy of the polymer samples was determined using the *sessile drop* technique with a home-made contact angle system (having a 2X lens goniometer equipped with a 2Mp digital camera, a cold LED light source, and a sample holder). Distilled water and glycerol drops of 1 μL volume (at room temperature, ∼23 °C) were placed onto the sample’s surfaces and the contact angle was recorded. The ImageJ software, v1.54d [30], with the Drop Analysis plug-in [31], was used for contact angle measurements. The surface energy ( γ ) was determined in our experiment using the polar and dispersive components of two liquids, distilled water and glycerol.

#### 2.2.3. Attenuated Total Reflectance Fourier Transform Infrared Spectroscopy

ATR-FTIR (attenuated total reflectance Fourier-transform infrared spectroscopy) is a powerful technique for the characterization of polymers. It enables the analysis of chemical structures of polymers by measuring the infrared absorption of a sample. This technique is advantageous because it can be used to study materials without the need for sample preparation, and can provide a wealth of information about the material’s molecular structure. The ATR-FTIR technique is based on the attenuation of infrared radiation as it passes through a sample. A prism is used to direct the beam of infrared onto the sample and, as the beam passes through the sample, the intensity of the beam decreases. This attenuation is then measured, and the absorption spectra of the material are calculated. The absorption spectra can then be used to identify the chemical structures of the polymer. The ATR-FTIR technique is used in a variety of fields, including polymer research, pharmaceutical analysis, and forensic science. It is especially useful for the characterization of polymers, as it can provide information on the chemical composition and structure of the polymer. For example, it can be used to identify and characterize the type of monomers used to synthesize the polymer, as well as the degree of cross-linking between them. Overall, ATR-FTIR is a powerful and versatile technique for the characterization of polymers. It is a non-destructive and reliable technique that can provide a wealth of information about the structure and chemical composition of a polymer [32,33,34,35,36].

FT-IR spectra were recorded with an FT/IR-4000 series spectrometer (Jasco, Tokyo, Japan) at room temperature with a resolution of 2 ${\mathrm{cm}}^{-1}$ in the range of 500–4000 ${\mathrm{cm}}^{-1}$ . Using the ATR-FTIR spectroscopy, the polymer samples prior to (the filaments) and after 3D printing (cuboid objects with a surface area of 10 × 10 ${\mathrm{mm}}^{2}$ and a thickness of 0.5 mm were employed) were analyzed.

#### 2.2.4. Broadband Dielectric Spectroscopy (BDS)

Broadband dielectric spectroscopy (BDS) is an important technique in the characterization of polymers, offering valuable insights into the physical and chemical properties of the material. These measurements are based on the material’s capacity to store electrical energy, allowing for the determination of key parameters such as dielectric permittivity, dielectric loss, and other relevant characteristics. BDS facilitates the exploration of molecular dynamics, phase transitions, and the mobility of charge carriers across various materials, encompassing polymers and polymer composites [37,38,39].

The broadband dielectric spectroscopy (BDS) measurements were conducted with a broadband dielectric spectrometer from Novocontrol Technologies, Montabaur, Germany. The samples were placed between two gold-plated flat electrodes and were inserted into the active sample cell test interface (ZGS) extension of the BDS device. The measurements were carried out under a dry nitrogen atmosphere to prevent moisture interference. The temperature was controlled with the Quatro Cryosystem device (also supplied by Novocontrol), and the samples were subjected to heating in the presence of a pure nitrogen flow, thereby preventing moisture from the environment. The WinDETA software provided by Novocontrol was used for BDS data acquisition. The dielectric data were collected under isothermal conditions across a frequency range of 1 Hz to 1 MHz, spanning temperatures from −150 to 200 °C.

#### 2.2.5. Differential Scanning Calorimetry (DSC) Measurements—Thermal Transitions

Differential scanning calorimetry (DSC) is a crucial tool for characterizing polymers by measuring the difference in heat absorption or release between a sample and a reference material. It determines the thermal properties of polymers, such as melting point, glass transition temperature, and heat capacity. DSC is particularly useful for analyzing the crystallinity of polymers, which is crucial for determining performance and processing characteristics. DSC is based on the fact that polymers absorb or release energy during physical transitions, such as melting or glass transitions. It is particularly useful for analyzing amorphous polymers without a distinct melting point and can also analyze the effect of additives on the thermal properties of the polymer. Despite its advantages, DSC is limited to crystalline polymers and is not sensitive to small amounts of additives. However, it is limited to the study of crystalline polymers and is not very sensitive to small amounts of additives [40,41].

The DSC measurements were conducted on a 200 F3 Maia DSC device (Netzsch, Waldkraiburg, Germany). Indium was used as a calibration standard sample. Around 11 mg of each sample was heated in aluminum crucibles with sealed shut pierced lids. The experiments were performed at a heating rate of 10 °C $\min^{-1}$ and in nitrogen as a purge gas (flow rate 50 mL $\min^{-1}$ ).

#### 2.2.6. X-ray Diffraction

X-ray diffraction (XRD) is an invaluable tool for characterizing polymers. It is an analytical technique that is used to identify and analyze the crystal structure of materials such as polymers on a nanometer scale. XRD uses X-ray radiation to probe the structure of polymers by measuring the diffracted X-ray intensity of a sample as a function of angle. The resulting diffraction pattern can be used to understand the structure and composition of the polymer, which in turn gives insight into the properties of the material. XRD is a technique that is widely used for characterizing polymers and has been successfully applied to materials such as polyethylene, polypropylene, polystyrene, polyamides, and polyurethanes, as well as polymers containing metallic elements. XRD measurements have been used to determine the degree of crystallinity, the crystallite size, the orientation of the crystallites, the crystal structure, and the molecular structure of the polymer. Furthermore, XRD can be used to detect and quantify impurities that may be present in the polymer. XRD is a non-destructive technique and can be used to analyze polymers in both their solid and liquid states. Its ability to provide detailed information about the structure of a polymer makes it an attractive choice for polymer characterization [42,43].

The crystalline structure of polymer samples was examined using an X-ray diffractometer (Rigaku Miniflex 600 diffractometer) with a Cu K α to 50.0° radiation ( λ = 1.54 Å), in the 2 θ range from 5.0 in steps of 0.01° and a recording rate of 1°/min.

## 3. Results and Discussion

This section has been divided into subheadings that are associated with plasma diagnosis, surface characterization, and a discussion of the data that were obtained. To be more specific, it offers a clear and precise account of the outcomes of the experiment, as well as their interpretation and the conclusions drawn from the experiment.

### 3.1. Plasma Source Electro-Optical Characterization

The methods for diagnosing plasmas at atmospheric pressure usually involve, for the basic study, the monitoring as well as the recording for further statistical processing of electro-optical quantities [18,19,20,21,22,23,44]. For electrical diagnosis, such quantities are represented by: the amplitude of the electric voltage applied to the discharge electrodes, the intensity of the discharge current on the grounding line, the calculation of the total charge deposited by the plasma on the surface with which it comes into contact, the estimation of the average power, and the energy transmitted by the plasma of the sample with which it interacts. For the optical diagnosis of plasma, the important quantities to be monitored and recorded are usually the total light emitted by the plasma, thus identifying the excited species in the plasma, which have physico-chemical potential to initiate changes to the surface it comes into contact with.

#### 3.1.1. Plasma Jet Electrical Diagnosis

**Figure 2:**
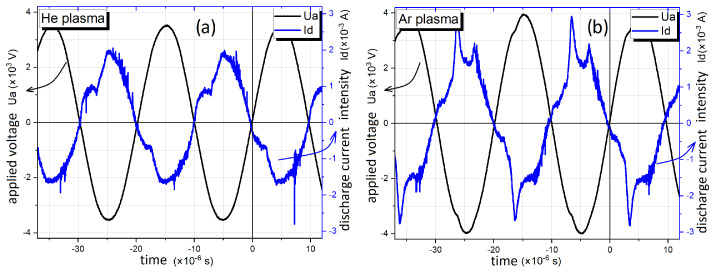
The voltage–current waveform of the discharge being studied and used for polymer treatment, ignited in (**a**) He and (**b**) in Ar.

The plasma’s basic electric diagnostic was related to the voltage that was applied, the discharge current, the monitoring of the charge, and the calculation of power and energy. Typical waveforms of voltage and current, for plasma running in both helium (left graph) and argon (right graph), are shown in Figure 2, which can be found below. These voltage–current representations are similar to those obtained in previous experiments, conducted in appropriate conditions [18,20].

**Figure 3:**
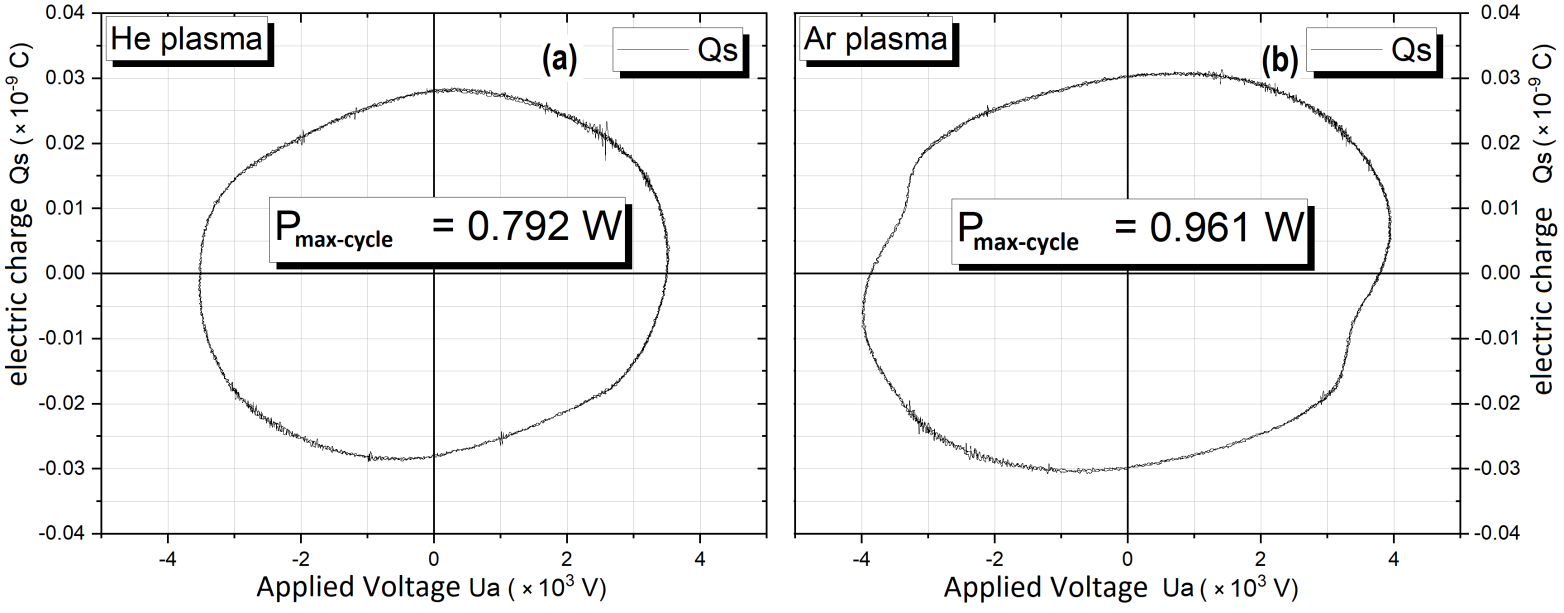
The charge–voltage waveform, Lissajous figure, of the discharge being studied, ignited in (**a**) He and (**b**) in Ar, used for power estimation.

The electrical power, calculated based on the area of Lissajous figures, ranged from 0.25 to 0.79 W, with a corresponding energy range of 5.5 to 17.4 μJ, for He plasma, respectively, from 0.3 to 0.96 W, with a corresponding energy range from 6.6 to 21.1 μJ, for Ar plasma, as presented in Figure 3.

#### 3.1.2. Plasma Jet Optical Diagnosis

**Figure 4:**
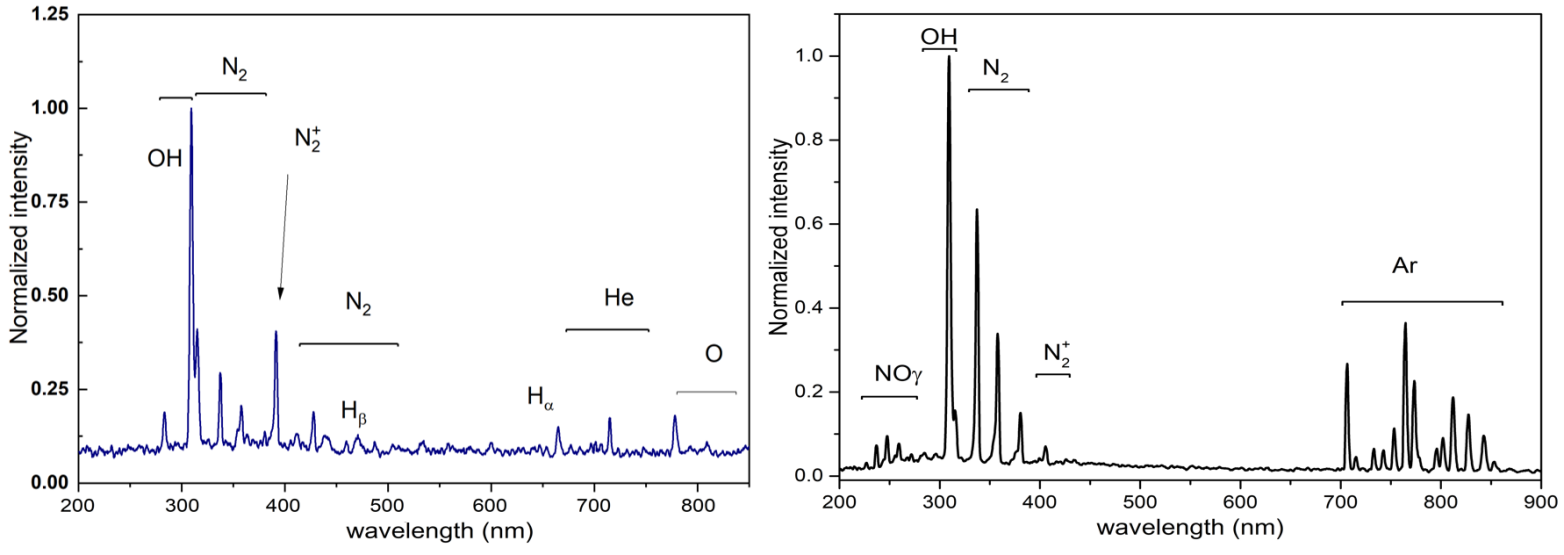
The optical emission spectra of the discharge being studied and used for polymer treatment.

Figure 4 illustrates that the plasma source utilized in this investigation possesses sufficient energy to stimulate not only the working gas lines, but also the lines and bands of other atmospheric species, including ${\mathrm{NO}}_{\gamma }$ , OH, $N_{2}$ , $N_{2}^{+}$ , and O, in addition to helium or argon lines. The reactive species of nitrogen and oxygen (RNS and ROS), commonly referred to as such, have the potential to exert a substantial influence on the interaction between the plasma and the sample surface.

In the wavelength range of 200 to 300 nm, the emission spectra resulting from the interaction of the Ar discharge with the polymer were primarily composed of the ${\mathrm{NO}}_{\gamma }$ lines, specifically at wavelengths of 237, 247, 259, and 271 nm. The hydroxyl radical (OH) rotating band was observed at a wavelength of 309 nm in both the helium (He) and argon (Ar) discharges. The molecular nitrogen ( $N_{2}$ ) bands were observed at wavelengths of 315 to 390 nm, namely at 315, 337, 357, and 375 nm. The $N_{2}^{+}$ molecular ion band was observed at a wavelength of 391 nm, which is crucial for determining gas temperature using the Boltzmann plot technique. The molecular nitrogen bands reappeared within the wavelength range of 400 to 470 nm. The gas lines used for work were measured in the range of 580–740 nm, specifically at wavelengths of 588, 668, 706, and 727 nm. Huzum et al. [18] and Nastuta et al. [19] have reported that the production and stimulation of the $N_{2}$ second positive nitrogen system (SPS) and the $N_{2}^{+}$ first negative nitrogen system (FNS) are dependent on the Penning effect of He metastables. In addition, the spectral lines of atomic oxygen were detected at wavelengths of 777 and 845 nm, which correspond to specific metastable states (triplet states) of oxygen. The OH and O bands and lines observed in the emission spectra are the result of the dissociation of ambient $O_{2}$ and $H_{2}$ O, as documented by previous studies [18,19,21,22,23,44] conducted under comparable plasma circumstances. The coexistence of reactive oxygen and nitrogen species with helium or argon lines, which are the working gases, indicates that these species are likely to actively participate in the treatment of wheat seeds. Furthermore, these excited species are significant because they can be utilized to determine the gas temperature and vibrational temperature. These spectroscopic temperatures offer valuable insights into the energetic species that will be employed in the sample treatment.

### 3.2. Surface and Volume Polymeric Sample Characterization Methods

#### 3.2.1. Surface Morphology: AFM and SEM

**Figure 5:**
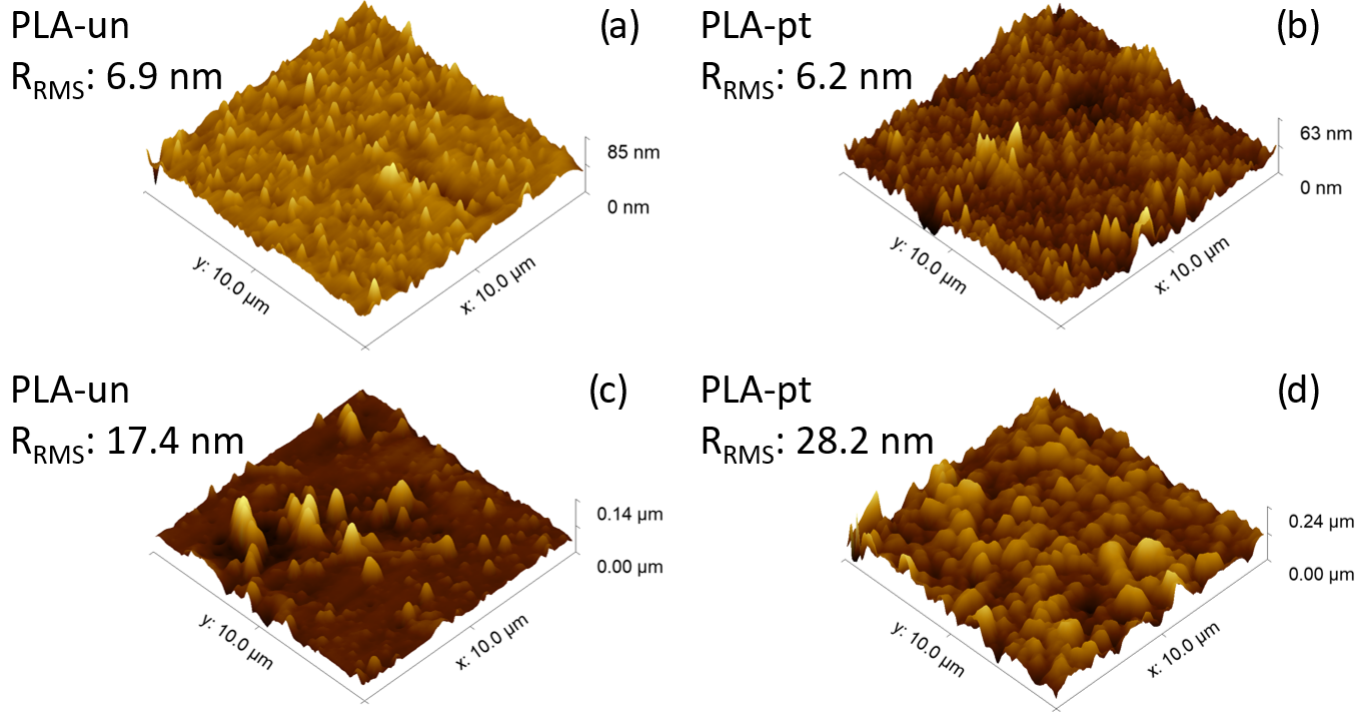
Three-dimensional (3D) topography images of untreated (**a**) and plasma treated filaments (**b**), respectively, images of 3D-printed untreated (un) (**c**) and plasma treated (pt) polymer (**d**).

AFM morphology pictures were obtained for each sample using a 10 µm × 10 µm scanning region. The imaging process was performed at various locations on the polymer sample to ensure the reproducibility of the data. The obtained results are primarily limited to three-dimensional photographs of the original and modified polymer surfaces (Figure 5). Statistical atomic force microscopy (AFM) estimations allowed for the characterization and comparison of surface roughness using the root mean square roughness ( $R_{RMS}$ ), both before and after the treatment. The root mean square roughness ( $R_{RMS}$ ) of pristine PLA filaments is 6.9 nm, whereas for the pristine 3D printed filaments it is 17.4 nm. The $R_{RMS}$ value for the plasma-treated filament sample is 6.2 nm, while for the plasma-treated and 3D-printed sample it is 28.2 nm. By examining the 3D images in Figure 5, it is evident that the morphology and roughness of the PLA samples have undergone noticeable alterations, both after plasma treatment, 3D printing, and the combination of these two processes. This phenomenon may be attributed to the reorganization mechanisms of PLA chains taking place at the surface of the polymer, maybe accompanied by crosslinking or bond cleavage reactions. The plasma exposure induces a ’smoothing’ process and uniform reorganization of the peaks on the surface, which can improve the contact angle between PLA and any gas, liquid, or solid it comes into contact with. This has the potential to boost adhesion for the surfaces treated with plasma. In order to support this assumption, it is necessary to employ further characterization techniques such as FTIR, SEM, CA, or XPS.

Because of this, a great number of features tend to increase on the surface. Naturally, these impacts can also be observed in the rise in the $R_{RMS}$ value, which is also dependent on the amount of time that the plasma is exposed to it.

Scanning electron microscopy (SEM) was used in order to follow the internal structure of the polymer that was being investigated (both in its natural state and after being treated in plasma) in much greater detail. In this sense, the polymer samples were fractured in liquid nitrogen and then their cross-sections were examined using an SEM. The investigated samples were coated with a thin layer(≈3 nm) of platinum using a Leica EM ACE200 Sputter coater to render electrical conductivity and to obstruct charge build-up during exposure to the electron beam.

**Figure 6:**
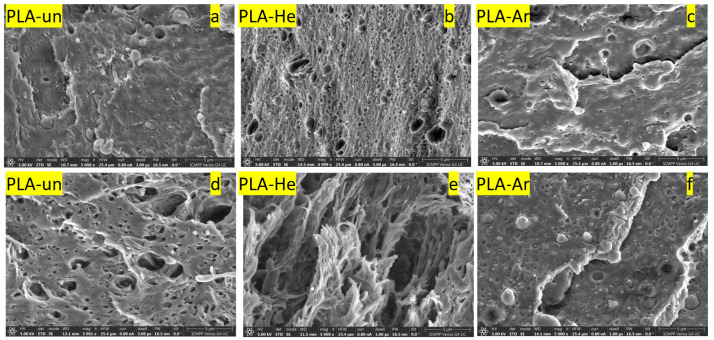
SEM images of the PLA samples cross-sections after liquid nitrogen fracture (**a**–**c**) and after mechanical stretching (**d**–**f**), for untreated (un) and plasma exposure (in He or Ar).

Figure 6 shows the cross-section micrographs for the PLA samples before and after the mechanical stretching tests. Thus, it is possible to observe the plasma treatment effect on the morphology and the way in which the plasma treatment affects the mechanical properties of the samples. From the SEM micrographs, it can be seen that the morphology of the samples varies depending on the type of plasma treatment. The untreated sample (PLA-un) shows a cross-section with small pores. This porosity is higher in the case of the He plasma-treated PLA (PLA-He) sample, where the presence of pores is much more obvious. In the case of the Ar plasma treated PLA (PLA-Ar) sample, minor changes are observed regarding the roughness or porosity of the material. The micrographs of the samples that were subjected to the mechanical stretching test are in correlation with the changes induced by the plasma treatment. Thus, PLA-He samples are the ones that show deformations of the internal structure of the pores and a certain orientation induced after the mechanical tests.

#### 3.2.2. Static CA and Surface Energy

To assess the wettability characteristics of the PLA surface, both before and after the plasma treatment, as well as before and after the 3D printing process, contact angle measurements were conducted using distilled water or glycerol on the polymer surface. This technique provides a rapid means of seeing the immediate effects of plasma therapy on surface changes. A homemade device, also used by [23], is employed to gauge and archive the photographs of liquid droplets. The polymeric sample was positioned between a microscope tube and a cold light source. Subsequently, droplets of distilled water and glycerol with a volume of 1 μ L were applied onto the polymer surface using a pipette with a capacity of 0.5–2.5 μ L. Each droplet is photographed under a microscope once equilibrium is reached between the droplet and the sample surface. The contact angle was calculated using the sessile drop technique. Unless explicitly specified, every measurement was conducted on a fresh location. The data points correspond to the mean value obtained from a minimum of three separate measurements, with a standard deviation of up to ±2°.

**Figure 7:**
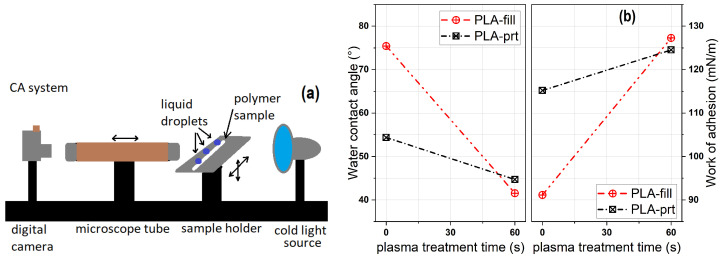
The contact angle set-up (**a**) and the values of the CA and work of adhesion for the PLA samples, untreated and He plasma treated, both in the filament (fill) and the printed (prt) state (**b**).

The contact angle set-up used in these studies (a) and the values of the CA and work of adhesion for the polymer samples (b) as seen in Figure 7. The surface free energy ( γ ) was determined using the polar and dispersive components of two liquids, in our experiment distilled water and glycerol, for which at 23 °C, $\gamma_{water}$ = 72.80 mN/m, respectively, $\gamma_{glycerol}$ = 64 mN/m. In Table 1, the contact angle values, work of adhesion, and surface free energy, as well as the disperse and polar index, are presented.

The contact angle value for the untreated PLA filament sample is 75° using distilled water, and 78° using glycerol. After He plasma treatment, the contact angle decreases down to 41° for water and down to 55° for glycerol, respectively, after Ar plasma treatment CA decreases to 46° for water and to 64° for glycerol (as in Figure 7). This is a direct proof of surface functionalization. The same tendency—but with a moderate decreasing CA value for both liquids—for the 3D-printed polymer samples was observed, as depicted in Table 1.

#### 3.2.3. ATR-FTIR Spectroscopy

**Figure 8:**
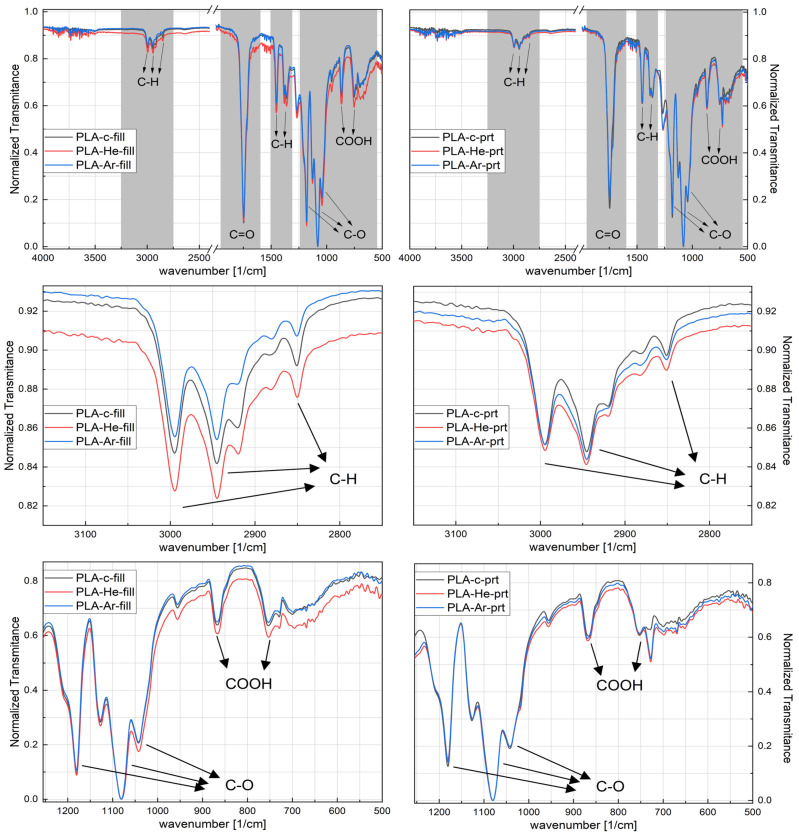
ATR-FTIR spectra of the polymer samples, on the left side filament (fill) and on the right side the printed (prt) samples.

The enhanced hydrophilicity of the plasma-treated filament and the 3D-printed samples can also be observed using Fourier transform infrared spectroscopy (ATR-FTIR). The ATR-FTIR spectrum for virgin PLA and He and Ar plasma-treated PLA is depicted in Figure 8. The spectra are shown for both the filament (‘fill’ extension in the left column of Figure 8) form and after 3D printing (‘prt’ extension in the right column of Figure 8). The presence of a peak at 1080 ${\mathrm{cm}}^{-1}$ can be attributed to the bonding between oxygen and carbon in the form of an oxygen–carbon–oxygen group. The peak seen at 1181 ${\mathrm{cm}}^{-1}$ corresponds to the stretching of –CH–O bonds. The signal observed at 1265 ${\mathrm{cm}}^{-1}$ corresponds to the C–O bonding of the ester group present in PLA. Furthermore, there is a distinct and prominent signal observed at 1745 ${\mathrm{cm}}^{-1}$ , indicating the presence of a carbonyl group (C=O). Upon plasma treatment with helium (He) and argon (Ar), the presence of oxygen-containing bonds, such as –O–C=O, –CH–O, C=O, and C–O, exhibited a clear correlation with the quantity of oxygen bonds. When ions, atoms, and excited molecules come into contact with the surface of PLA, they erode the surface and cause the polymer chains to break. These sites initiate a chemical reaction with air to generate molecules that include oxygen. Furthermore, the presence of absorption peaks within the range of 3650 to 3300 ${\mathrm{cm}}^{-1}$ , at 950 ${\mathrm{cm}}^{-1}$ and 832 ${\mathrm{cm}}^{-1}$ , together with a heightened peak at around 1370 ${\mathrm{cm}}^{-1}$ following treatment, can likely be ascribed to the stretching vibrations of OH groups, which contribute to the enhanced hydrophilicity of the samples. Similar results were also found by Tan et al. [45] and Zarei et al. [46,47] in appropriate experimental conditions.

According to Vasanthan et al. [48], in the wavenumber region between 600 and 1000 ${\mathrm{cm}}^{-1}$ , there are the bands corresponding to the crystalline (around 920–921 ${\mathrm{cm}}^{-1}$ ) and amorphous state of PLA (≈955–956 ${\mathrm{cm}}^{-1}$ ). Moreover, Vasanthan also monitored some band in the 2800–3200 ${\mathrm{cm}}^{-1}$ (2996, 2961 and 2945 ${\mathrm{cm}}^{-1}$ ) that have evolutions related to the polymer crystalline state. It was observed that the helium plasma treatment of PLA induced the most pronounced effect to be seen in the FTIR spectra. As expected, due to the fact that the PLA filament was subject to plasma exposure and after that subjected to the 3D printing process, the effects were pronounced for the spectra obtained for it; as can be seen in Figure 8 on the left side, from top to bottom, the full range and representative regions for the C–H, C–O, and COOH. The main hypothesis here is that, after plasma exposure, the surface of the PLA comes into contact with more reactive oxygen species, so slight modifications of the C–O and COOH regions are to be encountered. Nevertheless, some modifications are also observed for the PLA exposed to argon plasma, but with less intensity. Nonetheless, these results suggest that, for the conditions presented in this report, plasma treatment mostly affects the surface rather than the bulk of the material. Furthermore, the 3D printing process may be seen as an annealing process, leading to an augmentation in the degree of crystallinity of the polymers. However, the plasma treatments continue to have noticeable effects even after the polymer is printed, indicating that the plasma effects are quite persistent during the 3D printing process.

#### 3.2.4. Dielectric Properties

**Figure 9:**
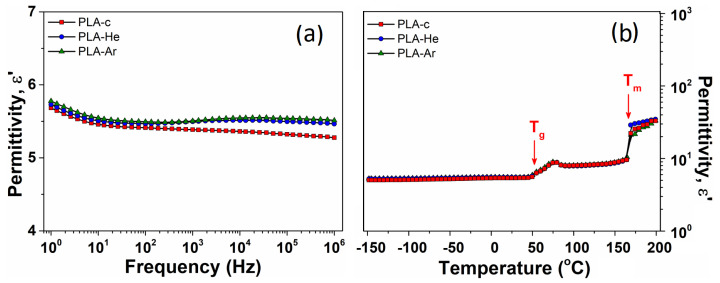
The evolution of dielectric permittivity with frequency at room temperature for untreated PLA-c and plasma treated PLA-He, PLA-Ar samples (**a**). To the right, the evolution of dielectric permittivity with a temperature at 1 kHz for untreated PLA-c and plasma treated PLA-He, PLA-Ar samples (**b**). The $T_{g}$ and $T_{m}$ points are marked with arrows.

The broadband dielectric spectroscopy (BDS) measurements were conducted across temperatures ranging from −150 to 200 °C. The behavior of dielectric permittivity ( $\epsilon^{\prime }$ ) with a frequency at room temperature for the 3D-printed PLA series is presented in Figure 9. The $\epsilon^{\prime }$ dielectric parameter exhibits a gradual decrease with increasing frequency, as a typical behavior for polymer materials [37]. The magnitude of $\epsilon^{\prime }$ is relatively high, as seen in the PLA-c sample, where f = 103 Hz, $\epsilon^{\prime }$ = 5.4, indicating a significant dipolar activity.

This outcome aligns with expectations due to the presence of numerous polarizable units in the chemical structure of PLA, as detailed in the FT-IR section. These units can effectively respond to the alternating electrical field, contributing to the dipolar signal of $\epsilon^{\prime }$ . It can be noted that the $\epsilon^{\prime }$ size of the plasma-treated samples is notably enhanced across the entire frequency spectrum, suggesting a slightly superior dipolar activity as compared to that of PLA-c. The numerical values of $\epsilon^{\prime }$ at room temperature and a frequency of 1 kHz are provided in Table 2.

Figure 9b displays the temperature-dependent variations of $\epsilon^{\prime }$ at a frequency of 1 kHz for the PLA series. At low temperatures, there is a slight increase in $\epsilon^{\prime }$ as temperature increases. During this regime, the thermal energy absorbed by polarizable units is limited, resulting in a restricted number of dipoles, such as polarizable units from side-chain macromolecules, being able to follow the alternating electrical field [49]. Beyond 50 °C, a distinct rise in $\epsilon^{\prime }$ is observed, corresponding to the glass transition temperature of PLA. Around $T_{g}$ , the polarization is noticeably enhanced due to thermal activation of macromolecule backbone. As the temperature continues to increase, a second-step increase of $\epsilon^{\prime }$ is noted, aligning with the melting point of PLA material, as indicated by DSC thermograms. The dielectric permittivity magnitude is slightly enhanced for plasma-treated samples across most of the temperature range, except after the melting point ( $T_{m}$ ) of PLA. However, it is essential to consider that the material melting may influence BDS measurements in this specific region.

**Figure 10:**
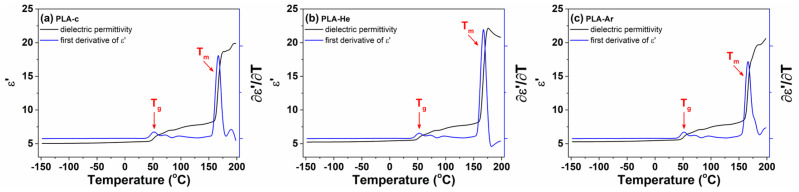
The evolution of dielectric permittivity and the first derivative of permittivity versus temperature for simple PLA-c (**a**) and treated PLA-He (**b**) and PLA-Ar (**c**) samples. The dielectric spectra are selected at 1 MHz. The $T_{g}$ and $T_{m}$ points are marked with arrows.

The changes in $\epsilon^{\prime }$ with temperature and the first derivative of dielectric permittivity are further represented in a common diagram in Figure 10 for the PLA series. In this arrangement, the thermal $T_{g}$ and $T_{m}$ events manifest as distinct step increases in the isochronal plots of $\epsilon^{\prime }$ versus temperature and as clearly defined dielectric peaks in the corresponding temperature dependencies of $\frac{\partial \epsilon^{\prime }}{\partial T}$ .

**Figure 11:**
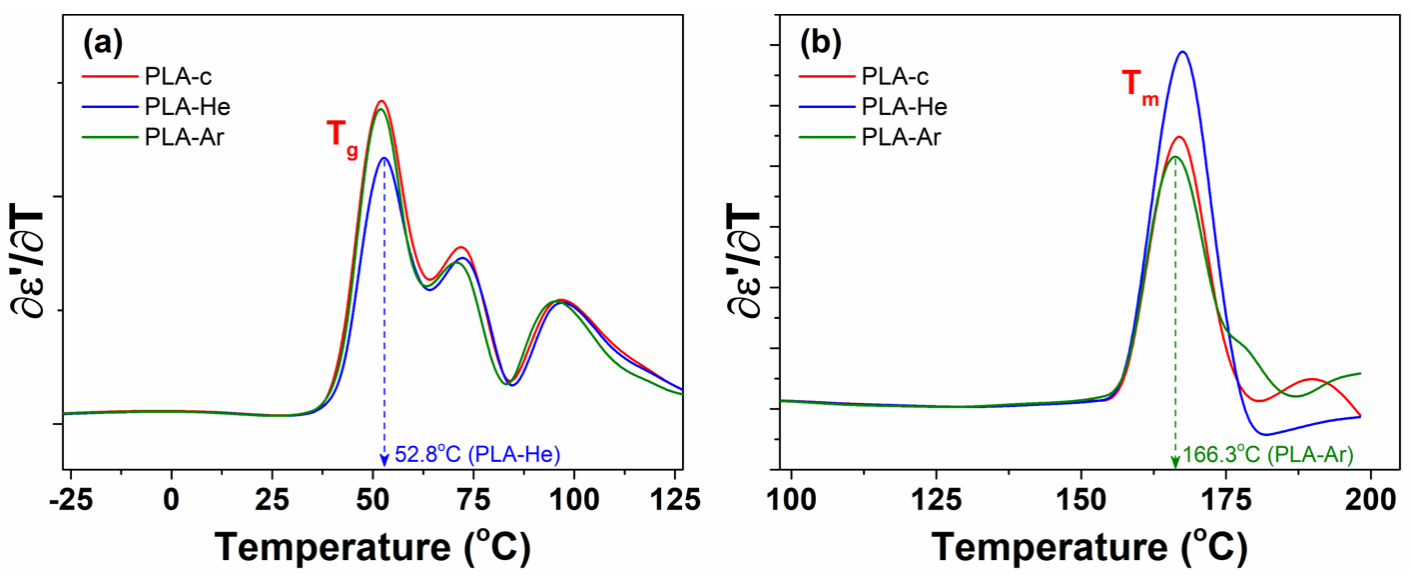
First derivative of permittivity versus temperature for simple PLA-c, PLA-He, and PLA-Ar in the temperature regions of $T_{g}$ (**a**) and $T_{m}$ (**b**). The arrows indicate the numerical values of $T_{g}$ and $T_{m}$ exemplarily for PLA-He and PLA-Ar, respectively.

The first derivatives of dielectric permittivity with respect to temperature are comparatively depicted in Figure 11 for the PLA series in the temperature regions corresponding to $T_{g}$ (a) and $T_{m}$ (b) transitions. $T_{g}$ is associated with the highest intensive dielectric peak observed in Figure 10, while the other signals result from supplementary temperature fluctuations of $\epsilon^{\prime }$ . The numerical values of $T_{g}$ and $T_{m}$ are detailed in Table 2 and correspond to the temperatures at which the peak maxima occur. The $T_{g}$ and $T_{m}$ values for the PLA series exhibit considerable similarity, suggesting that the effects of plasma treatment on thermal $T_{g}$ and $T_{m}$ events are not distinctly discernible through BDS measurements.

#### 3.2.5. Differential Scanning Calorimetry (DSC)

**Figure 12:**
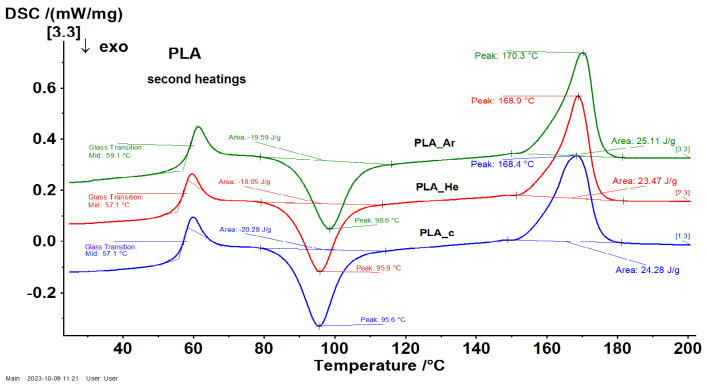
Representative DSC thermograms for the series of PLA during the second heating at 10 °C $\min^{-1}$ .

DSC measurements were performed over two heating regimes and a cooling phase. The first heating regime was performed for erasing the thermal history of samples within the temperature range of 25 °C to 200 °C. Subsequently, the samples underwent a cooling process from the melt state to 25 °C. Finally, a second heating cycle was performed, spanning temperatures from 25 °C up to 200 °C. Figure 12 here presents the DSC curves of the series of 3D-printed PLA samples, during the second heating cycle of measurements. Notably, no exothermal peaks, such as crystallization, were detected during the cooling phase, indicating that all the investigated samples remained entirely amorphous. Following the second heating regime, the main thermal events for the untreated PLA-c sample were identified. These include the glass transition step ( $T_{g}$ ) at approximately 51.6 °C, the cold crystallization exothermic peak ( $T_{cc}$ ) at 103.7 °C, and a two-step melting process ( $T_{m}$ ) at 148.7 °C and 164.9 °C. The temperature values associated with these thermal processes during the second heating cycle for all investigated samples are detailed in Table 3. It is noteworthy that the thermal processes, namely $T_{g}$ , $T_{cc}$ , and $T_{m}$ , for plasma-treated samples occurred at higher temperatures compared to those of the untreated PLA sample.

#### 3.2.6. X-ray Diffraction

X-ray diffraction analysis was also carried out in order to evaluate the phase crystallization and chemical composition of various PLA objects that were manufactured using 3D printing technology. The creation of the amorphous phase of the polymer is demonstrated by a large peak in pure PLA, which is positioned at the diffraction angle of around 16°, as reported by [46,50].

**Figure 13:**
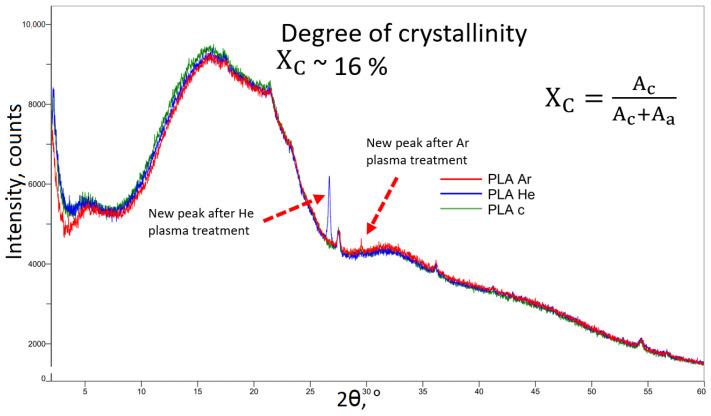
XRD spectra of the studied 3D-printed polymer samples: untreated (c), He, and Ar plasma-treated PLA.

A critical parameter for polymers being processed, by every means, is the degree of crystallinity (or crystallization capacity) $X_{c}$ , which is estimated from the difractograms of the polymeric samples. For pure PLA samples, the $X_{c}$ is around 2.5%, as also reported in [46,50], and it increases after plasma treatment up to 16%, as seen in Figure 13.

This peak is illustrated in Figure 13. After plasma exposure of the samples, it can be hypothesized that the PLA crystal peak became more narrow and sharper as a result of the crystallinity augmentation of PLA, which was in accordance with the results of the DSC.

Additionally, new crystalline peaks were found in the diffractograms that were acquired after the treatment carried out in He plasma at an angle of 26° and after the treatment carried out in Ar plasma at an angle of 30°.

## 4. Conclusions

The analysis of the plasma sources under study indicates the operational parameters for surface treatment, as determined using electrical and optical diagnostic techniques.

After conducting research on surface morphology, it was shown that plasma treatments led to significant reshaping of the surfaces, resulting in the production of evenly dispersed nanometric structures.

Surface wettability is determined by the combined influence of surface roughness and chemical characteristics. The enhanced hydrophilicity of the surface is seen by an elevation in surface energy following plasma exposure.

The results indicate that electric discharges in gases at atmospheric pressure are a suitable and adaptable method for altering the surfaces of materials, for specific applications.

The XRD data exhibited minor variations, with the exception of a few more peaks, which can be linked to a slight elevation in the glass temperature observed in the DSC measurements.

Based on BDS measurements, it can be observed that the $\epsilon^{\prime }$ magnitude of the plasma-treated samples is significantly increased across all frequencies. This indicates a slightly higher dipolar activity relative to the control polymers. Furthermore, the magnitude of dielectric permittivity is marginally increased for samples treated with plasma across the majority of the temperature range.

Additionally, it is necessary to conduct experiments to effectively regulate the surface alterations resulting from plasma treatment. These experiments should involve techniques such as X-ray photoelectron spectroscopy (XPS), 3-point bending and impact tests, and ink testing, as well as scratch and pull-off tests.

## Figures and Tables

**Table 1:** The values of the PLA contact angle, work of adhesion and free energy.

		Contact Angle		Work of Adhesion		Surface Free Energy			$x^{d}$ ^1^	$x^{p}$ ^2^
		(°)		( ${\mathrm{mN/m}}^{2}$ )		( ${\mathrm{mN/m}}^{2}$ )			(%)	(%)
Sample	Plasma ON (s)	Distilled Water	Glycerol	$W_{12}$ -Water	$W_{12}$ -Glycerol	γ	$\gamma^{d}$	$\gamma^{p}$	$\gamma^{d}$ / γ	$\gamma^{p}$ / γ
PLA-c-fill ^3^	0	75	77	91.13	77.34	30.80	2.73	28.06	0.088	0.911
PLA-He-fill	60	41	55	127.29	100.66	72.21	0.45	71.75	0.006	0.993
PLA-Ar-fill	60	46	64	107.78	91.62	36.03	3.19	32.83	0.087	0.911
PLA-c-prt ^4^	0	54	58	115.20	97.13	38.93	3.45	35.47	0.088	0.911
PLA-He-prt	60	44	48	124.53	106.32	70.64	0.44	70.19	0.006	0.993
PLA-Ar-prt	60	50	53	118.90	102.53	40.18	3.56	36.60	0.088	0.910

^1^ $x^{d}$ (%) = disperse index. ^2^ $x^{p}$ (%) = polar index. ^3^ fill = PLA fillament. ^4^ prt = printed PLA.

**Table 2:** Numerical values for dielectric permittivity and the thermal events assessed by BDS measurements.

Sample	BDS Measurements		
	$\epsilon^{\prime }$ (r.t. and f = 1 kHz)	$T_{g}$ (°C)	$T_{m}$ (°C)
${\mathrm{PLA}}_{c}$	5.4	52.2	166.8
${\mathrm{PLA}}_{He}$	5.5	52.8	167.5
${\mathrm{PLA}}_{Ar}$	5.5	52.0	166.3

**Table 3:** Numerical values for the thermal events assessed by DSC measurements.

Sample	$T_{g}$ (°C)	$T_{\mathrm{cc}}$ (°C)	$T_{m}$ (°C)	
			1	2
${\mathrm{PLA}}_{c}$	51.6	103.7	148.7	164.9
${\mathrm{PLA}}_{He}$	53.6	110.1	151.3	166.6
${\mathrm{PLA}}_{Ar}$	54.7	112.3	152.3	167.2

## Data Availability

Raw data may be available, on reasonable request, from the authors.
